# Multidimensional perceived school support and AI learning anxiety among university students: the mediating roles of control-value appraisals

**DOI:** 10.3389/fpsyg.2026.1951992

**Published:** 2026-09-10

**Authors:** Hongyan Jiang, Bingquan Chen, Qiaoyun Chen

**Affiliations:** 1 School of Marxism, Nanjing Normal University, Nanjing, China; 2 School of Science, Nanjing University of Posts and Telecommunications, Nanjing, China; 3 School of Education Science, Nanjing Normal University, Nanjing, China

**Keywords:** AI learning anxiety, AI learning self-efficacy, control-value theory, multidimensional perceived school support, perceived usefulness, structural equation modeling (SEM)

## Abstract

**Introduction:**

As artificial intelligence (AI) becomes embedded in university learning, students may experience anxiety when they doubt their capacity to learn with it or question its academic value. This study examines the direct association between multidimensional perceived school support and AI learning anxiety and the indirect associations involving control-value appraisals, represented by AI learning self-efficacy and perceived usefulness.

**Method:**

Data from 547 Chinese university students were analyzed using structural equation modeling (SEM) to test direct, parallel indirect, and sequential indirect paths. Specific and sequential indirect paths were evaluated using 5,000 bootstrap resamples and 95% bias-corrected confidence intervals. Supplementary artificial neural network (ANN) analyses assessed predictive performance and relative predictor importance.

**Results:**

Greater perceived school support is associated with higher AI learning self-efficacy (β = 0.469, *p* < 0.01) and perceived usefulness (β = 0.223, *p* < 0.01), and with lower AI learning anxiety (β = −0.160, *p* < 0.01). Self-efficacy is positively associated with perceived usefulness (β = 0.587, *p* < 0.01). Both self-efficacy (β = −0.408, *p* < 0.01) and perceived usefulness (β = −0.345, *p* < 0.01) are associated with lower AI learning anxiety. Together, the indirect paths account for 69.5% of the total standardized association. ANN analyses identify self-efficacy and perceived usefulness as the strongest and most stable predictors of anxiety.

**Discussion:**

The findings characterize AI learning anxiety as an achievement-related emotion associated with students' perceived support and their control-value appraisals. Universities may therefore consider combining encouragement, clear guidance, and practical resources to support students' confidence and recognition of AI's academic value.

## 1 Introduction

Artificial intelligence (AI) has moved from a peripheral technology to a routine element of many university learning environments. It is now embedded in teaching, assessment, feedback, and learner services, making institutional decisions about access, guidance, and evaluation increasingly consequential for students' learning experiences (Zawacki-Richter et al., 2019; Crompton and Burke, 2023). Generative AI has made these decisions particularly visible by providing immediate assistance with writing, programming, inquiry, and problem solving (Kasneci et al., 2023; Lo, 2023). Yet its educational value depends on more than the availability of a tool. Students must learn when to rely on an output, how to check its quality, and how to use it without undermining their own learning or academic integrity (Chan and Hu, 2023; Farrokhnia et al., 2024; Corbin et al., 2025).

Students differ considerably in their emotional responses to AI-supported study. For some, AI offers useful learning opportunities; for others, its rapid development, uncertain output quality, and unfamiliar learning demands can evoke doubt, frustration, and anxiety (Wang and Wang, 2022; Karaoglan Yilmaz et al., 2025). AI anxiety has consequently received increasing attention in research on students' engagement with these technologies. Wang and Wang (2022) developed a measure covering several dimensions of anxiety toward AI, while subsequent studies link such anxiety with AI learning behavior and motivated learning (Wang Y.-M. et al., 2024; Chen et al., 2025). AI learning anxiety refers more specifically to tension and worry arising when students acquire AI knowledge, interpret AI functions, or develop AI-related capabilities (Wang Y.-M. et al., 2024; Chai and Sha, 2025). It differs from broader AI concerns because it centers on the demands of learning rather than societal, ethical, or employment consequences. Supportive conditions may be particularly relevant at this point: clear guidance and opportunities for successful use may be associated with more predictable and manageable AI learning experiences and lower anxiety (Chai and Sha, 2025).

AI learning anxiety therefore raises a broader educational challenge of helping students engage with AI critically, responsibly, and with confidence. Psychological literacy provides a useful framework for understanding how this challenge can be addressed. It is commonly defined as the adaptive and intentional application of psychological knowledge to personal, professional, and societal needs (Roberts et al., 2015; Cranney et al., 2022). This broader capability-based account includes critical thinking, ethical judgment, and reflective insight into one's own and others' behavior and mental processes (Roberts et al., 2015). Applied to AI-supported learning, psychological literacy involves more than critically evaluating AI-generated information. It also involves reflective engagement with one's own learning processes, including recognizing one's perceived control over AI-related tasks, judging AI's educational value, and responding constructively to uncertainty or anxiety that may arise. It also highlights the role of universities: beyond providing access to AI tools, they can offer guidance, opportunities for reflection and practice, and practical resources that help students engage thoughtfully with AI. Accordingly, psychological literacy provides an educational lens for examining how school support may relate to students' control and value appraisals and to their emotional experiences in AI-supported learning.

Much of the existing work treats anxiety as a predictor of attitudes, intentions, motivation, or learning behavior (Wang Y.-M. et al., 2024; Chen et al., 2025). Recent studies of its correlates have emphasized individual resources, including AI literacy, self-efficacy, digital competence, and self-regulation (Wu et al., 2026; Karaoglan Yilmaz et al., 2025). Internationally, studies from Turkey, South Korea, and Saudi Arabia likewise link AI anxiety to students' AI literacy and attitudes, evaluative learning practices, and learning motivation (Cengiz and Peker, 2025; Kim, 2026; Mohamed et al., 2026). This evidence is valuable for understanding the individual capacities involved, but less is known about how the educational environment relates to students' control and value appraisals and emotional experiences in AI-supported learning. Among vocational college students, teacher support has been associated with lower AI learning anxiety and higher AI self-efficacy (Chai and Sha, 2025). Perceived school support is broader than teacher encouragement, however, because it encompasses the institutional conditions surrounding AI learning: reassurance when students are uncertain, usable guidance about appropriate practices, and practical resources such as training, tools, and technical help. Examining these forms of support together can clarify how perceived school support is associated with students' AI-related emotional experiences.

The proposed model draws on control-value theory (CVT), social support theory, and the technology acceptance model (TAM). CVT links achievement emotions to learners' evaluations of control and value (Pekrun, 2006, 2024; Tze et al., 2022). In this study, AI learning self-efficacy captures perceived control over AI-related tasks, whereas AI perceived usefulness represents the value students assign to AI in learning. Social support theory informs the distinction between emotional, informational, and instrumental school support (House, 1981; Tardy, 1985). TAM, in turn, treats usefulness as the expectation that technology use will enhance task performance (Davis, 1989). Bringing these perspectives together enables us to examine the association between perceived school support and AI learning anxiety and the corresponding appraisal patterns represented in the structural model.

Within this broader educational psychology context, this study examines the direct association between multidimensional perceived school support and AI learning anxiety, as well as the specific and sequential indirect associations involving AI learning self-efficacy and AI perceived usefulness. It makes three contributions.

1. It shifts attention from individual characteristics toward the learning environment by examining perceived school support as a multidimensional correlate of AI learning anxiety. This approach considers how encouragement, guidance, and material resources are associated with students' emotional adjustment to AI-supported learning.
2. It examines the specific and sequential indirect associations involving AI learning self-efficacy and perceived usefulness, thereby connecting perceived school support, students' control and value appraisals, and AI learning anxiety within a theory-consistent structural model.
3. It evaluates this structural model among undergraduates enrolled at Chinese universities using structural equation modeling (SEM) and bootstrap confidence intervals, and supplements the structural association analysis with artificial neural network (ANN)-based out-of-sample prediction and predictor-importance estimates.

## 2 Theoretical background and hypothesis development

### 2.1 Construction of the theoretical framework

CVT serves as the principal framework. It proposes that achievement emotions reflect learners' judgments of how much control they have over activities and outcomes and how much value they place on them (Pekrun, 2006, 2024). Anxiety is likely when learners attach importance to a task but doubt that they can manage its demands or outcomes. Across educational settings, control and value appraisals have been useful for explaining anxiety, enjoyment, boredom, and other performance-related emotions (Tze et al., 2022; Putwain et al., 2018; Shao et al., 2020). AI learning makes both appraisals salient: students may value AI for current study and future development while feeling unsure about their ability to understand its functions, evaluate generated information, or use it appropriately.

In the present model, perceived school support is specified as a more distal contextual correlate of students' immediate appraisals. Its dimensions follow social support theory, which differentiates support by the content and function of the resources provided (House, 1981; Tardy, 1985). Perceived school support may be associated with less ambiguous AI learning experiences and stronger perceptions of control and value. This reasoning accords with evidence that educational support affects technology acceptance (Zheng et al., 2018). Research in Chinese mathematics classrooms also associates teacher support with achievement emotions through academic control and value, illustrating its role as a contextual input to control-value processes (Chen and Leung, 2023).

TAM complements CVT by explaining perceived usefulness. In TAM, usefulness is the belief that using a technology will improve performance (Davis, 1989). Students do not necessarily develop this belief just because an AI tool is available; they require guidance, feedback, and opportunities to observe how AI affects efficiency, task quality, and academic work. TAM therefore helps account for links from perceived school support and AI learning self-efficacy to perceived usefulness.

### 2.2 The Association between school support and AI learning anxiety

Social support theory suggests that support can be distinguished according to the content and function of the resources provided, such as emotional, instrumental, informational, and appraisal-related support (House, 1981; Tardy, 1985). Context-specific studies also adapt these support dimensions to the source and setting of support, such as perceived organizational family support (Jahn et al., 2003). Focusing on university support for AI learning, we conceptualize perceived school support through three dimensions that are especially relevant to students' AI-related learning experiences: emotional support, informational support and instrumental support. Emotional support refers to encouragement, care, and acceptance that help students cope with uncertainty in AI learning. Informational support refers to guidance that helps students understand how to use AI appropriately, evaluate AI-generated outputs, and meet academic expectations. Instrumental support refers to access to AI devices, auxiliary tools, digital resources, and maintenance support.

Student adjustment is consistently associated with emotional, informational, and instrumental support (Malecki and Demaray, 2003), and research on academic emotions similarly indicates that supportive teaching conditions are associated with more favorable emotional experiences (Federici and Skaalvik, 2014; Lei et al., 2018; Chen and Leung, 2023). AI learning may be experienced as anxiety-provoking when students are unsure how to operate a tool, judge the quality of generated material, or follow appropriate-use norms. In this context, emotional support may be associated with a lower sense of facing these difficulties alone, informational support with clearer expectations for competent use, and instrumental support with more accessible opportunities for practice and help-seeking. Research on perceived teacher support similarly links stronger support to lower anxiety through self-efficacy (Wang C. et al., 2024); AI-specific work also links teacher support to lower AI learning anxiety (Chai and Sha, 2025). Extending this evidence beyond the classroom, stronger perceived school support is expected to be associated with a lower tendency to view AI learning as uncertain or threatening. Hence:

**H1**. *Perceived school support negatively predicts AI learning anxiety*.

### 2.3 The mediating role of AI learning self-efficacy

Self-efficacy refers to individuals' confidence about their capacity to plan and carry out the actions required to attain desired outcomes (Bandura, 1977, 1997). Although self-efficacy is not identical to control appraisal, task-specific self-efficacy captures students' judgment of whether they can take the actions needed to cope with task demands successfully. It can therefore be understood as an important expression of perceived control in AI learning. AI learning self-efficacy denotes students' perceived capability and confidence in learning AI-related knowledge and skills, including their belief that they have the ability, foundation, and personal resources needed to handle AI learning tasks (Wang Y.-M. et al., 2024).

Perceived school support is expected to be positively associated with AI learning self-efficacy, given that it reflects access to practice opportunities, encouragement, and a more predictable learning environment. Its three forms correspond to reassurance, clearer strategies, and opportunities to practice with AI. Prior evidence links teacher support to information literacy through information and communication technology (ICT) self-efficacy and perceived usefulness (Chen and Ma, 2022); institutional support also predicts perceptions of AI learning through learning outcomes and technology self-efficacy (Jeilani and Abubakar, 2025). Recent research on AI tool adoption further identifies university support and self-efficacy as antecedents of perceived usefulness (Zhao et al., 2025), while student–generative-AI interaction has been linked to learning outcomes through self-efficacy (Liang et al., 2023). Together, these considerations support the expected positive association between perceived school support and confidence in handling AI learning tasks. We propose:

**H2**. *Perceived school support positively predicts AI learning self-efficacy*.

Stronger efficacy beliefs are expected to be associated with greater perceived control and lower worry about inadequate ability or task failure. Longitudinal evidence relates stronger self-efficacy to lower test anxiety (Roick and Ringeisen, 2017). In AI-supported learning, AI self-efficacy has been found to predict less general AI anxiety and to promote actual AI use indirectly through that reduction (Chen et al., 2024). Because general AI anxiety and AI learning anxiety are not identical, specific evidence is important. Chai and Sha (2025) reported that AI self-efficacy was associated with lower AI learning anxiety. Students with greater AI learning self-efficacy are therefore expected to report more control over AI-related learning and less anxiety about inability or failure. It should also carry part of the association between perceived school support and AI learning anxiety:

**H3**. *AI learning self-efficacy negatively predicts AI learning anxiety*.
**H4**. *AI learning self-efficacy mediates the relationship between perceived school support and AI learning anxiety*.

### 2.4 The mediating role of AI perceived usefulness

Within TAM, perceived usefulness is a user's expectation that a technology will lead to better performance, making it central to technology acceptance (Davis, 1989). Applied to AI-supported learning, it denotes the belief that AI can offer practical benefits by improving the efficiency or quality of task completion, productivity, and performance (Achmadi et al., 2025). This belief is more likely to develop when students understand where AI can assist their learning and can connect its use with concrete academic outcomes.

Perceived school support may create opportunities for students to recognize and test AI's learning value. In learning-management-system research, organizational support strengthened self-efficacy and technical support, which then increased perceived system benefits (Zheng et al., 2018). Recent higher-education research likewise reports positive associations of university support and self-efficacy with the perceived usefulness of AI tools (Zhao et al., 2025). Likewise, students who experience more support from their school should have more occasions to understand, use, and verify AI's practical contribution to learning. We therefore expect perceived usefulness to be higher:

**H5**. *Perceived school support positively predicts AI perceived usefulness*.

Perceived usefulness is expected to be negatively associated with AI learning anxiety because it reflects a more favorable value appraisal of AI-supported learning. When students expect AI to improve efficiency, task quality, or academic performance, AI learning is more likely to appear worthwhile and manageable rather than merely difficult or threatening. Consistent with this expectation, perceived AI usefulness was negatively related to educational anxiety in smart-education settings (Tian, 2026). Accordingly, AI perceived usefulness is expected to predict lower learning anxiety and to mediate the relationship between perceived school support and that anxiety:

**H6**. *AI perceived usefulness negatively predicts AI learning anxiety*.
**H7**. *AI perceived usefulness mediates the relationship between perceived school support and AI learning anxiety*.

### 2.5 Sequential mediation of AI learning self-efficacy and AI perceived usefulness

CVT identifies control and value appraisals as joint sources of achievement emotions, but AI learning may also allow one appraisal to support the other. AI learning self-efficacy reflects students' sense that they can manage AI-related tasks. Students who feel capable may explore functions more freely, persist through problems, and accumulate successful experiences. Those experiences can make AI's practical value more apparent and help students decide whether it improves efficiency, task quality, or academic performance. AI-adoption research similarly reports a positive association between AI self-efficacy and the perceived usefulness of AI-enabled technologies (Shao et al., 2025). We therefore expect greater AI learning self-efficacy to be associated with greater perceived usefulness:

**H8**. *AI learning self-efficacy positively predicts AI perceived usefulness*.

Together, H2, H6, and H8 specify a theoretically ordered sequential indirect association involving perceived school support, AI learning self-efficacy, perceived usefulness, and AI learning anxiety. This ordering reflects the proposed structural model rather than an empirically established temporal process. Thus, we propose:

**H9**. *AI learning self-efficacy and AI perceived usefulness sequentially mediate the relationship between perceived school support and AI learning anxiety*.

**Figure 1:**
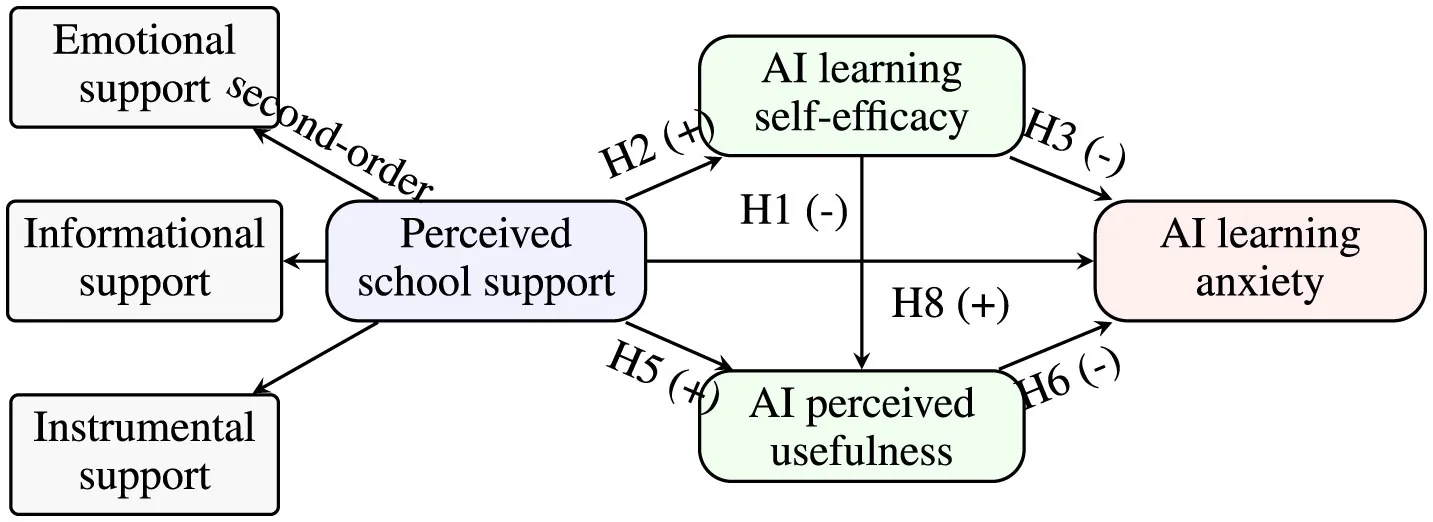
Research model and hypotheses.

Figure 1 depicts the hypothesized model. It shows the second-order structure of perceived school support and the proposed paths to AI learning self-efficacy, AI perceived usefulness, and AI learning anxiety.

## 3 Methodology

### 3.1 Participants and data collection

Participants were undergraduates enrolled at Chinese universities. Before the formal survey, two experts in relevant fields reviewed the questionnaire for construct relevance, clarity, content appropriateness, and overall structure. Their feedback led to minor revisions to the instructions and wording of some items without changing the substantive meaning of the original scales. A pilot study with 50 undergraduates similar to the target population then assessed item clarity, response-option appropriateness, completion time, online-survey functionality, and preliminary internal consistency using Cronbach's α. Given the limited pilot sample, construct validity was examined only with the formal-survey data; pilot responses were not included in the final analysis.

**Figure 2:**
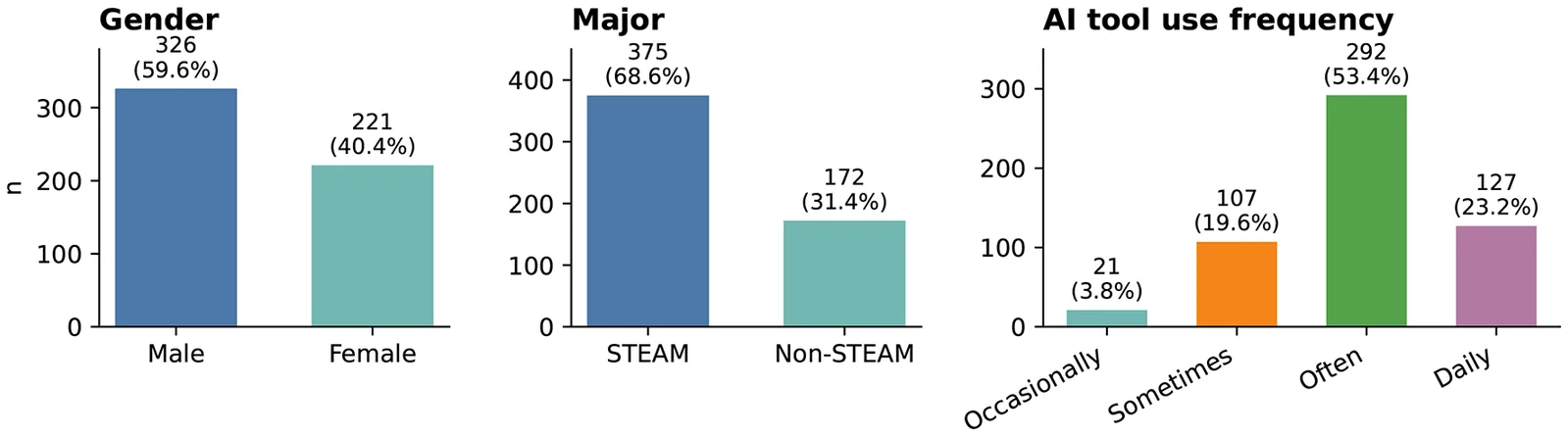
Sample composition of the valid responses.

Data were collected through a two-wave online survey administered on Wenjuanxing, with an interval of approximately 6 weeks between waves. Before the first wave, participants received an information statement explaining the study purpose, the voluntary nature of participation, the confidentiality of responses, and their right to withdraw at any time without penalty. No personally identifiable information was collected. The first wave gathered demographic information, AI learning self-efficacy, and AI perceived usefulness. The second wave assessed perceived school support and AI learning anxiety. A total of 634 questionnaire sets were returned across the two waves. Of these, 87 were removed because of implausibly short completion times, failed attention checks, or no experience using AI tools. The final analytic sample therefore comprised 547 responses, yielding an effective response rate of 86.3%. Table 1 shows that 326 respondents were male (59.6%) and 221 were female (40.4%). Science, technology, engineering, arts, and mathematics (STEAM)-related majors accounted for 375 students (68.6%), while 172 students (31.4%) studied in non-STEAM fields. AI use was already familiar to the sample: 53.4% reported frequent use and 23.2% reported daily use. Figure 2 provides a visual summary of the valid sample's gender, major, and AI-tool-use-frequency distributions.

**Table 1:** Sample characteristics.

Variable	Category	Frequency	Percentage (%)
Gender	Male	326	59.6
	Female	221	40.4
Major	STEAM	375	68.6
	Non-STEAM	172	31.4
AI tool use frequency	Occasionally	21	3.8
	Sometimes	107	19.6
	Often	292	53.4
	Daily	127	23.2

### 3.2 Measurement instruments

The survey contained 30 items covering perceived school support (PSS), AI learning self-efficacy (ASE), AI perceived usefulness (APU), and AI learning anxiety (ALA). The corresponding abbreviations are also provided in the Abbreviations section at the end of the manuscript.

#### 3.2.1 Perceived school support

Perceived school support was adapted from Feng et al. (2025)'s perceived organizational support scale for AI adoption. Although originally developed for workplace settings, its emotional, informational, and instrumental support dimensions are also applicable to AI-supported learning in universities, as they address students' needs for emotional encouragement, learning guidance, and access to necessary devices and resources. Accordingly, the original three-dimensional structure was retained, while workplace referents were replaced with school and student referents and the items were contextualized for university AI learning. The adapted scale comprised 13 items: five for emotional support (ES), four for informational support (IFS), and four for instrumental support (IS).

Content validity was evaluated through expert review and pilot testing. Two experts with expertise in educational technology and psychometrics reviewed the adapted items for construct relevance, clarity, contextual appropriateness, and coverage of the three dimensions. Their feedback led to minor revisions to the instructions and item wording without altering the intended meanings. A pilot test with 50 undergraduates subsequently assessed item clarity, response-option appropriateness, completion time, survey functionality, and preliminary internal consistency. Representative items were “When I face difficulties using AI technologies, my school shows empathy and understanding” for emotional support, “When using new technologies, my school provides guides or training materials” for informational support, and “My school provides appropriate devices for operating AI technologies” for instrumental support. Together, the expert review and pilot findings supported the content validity and suitability of the adapted scale for university students.

#### 3.2.2 AI learning self-efficacy

AI learning self-efficacy was assessed with the learning self-efficacy scale developed by Wang Y.-M. et al. (2024). Its original focus is students' confidence in learning AI-related skills, so no item wording was changed. The five items assess perceived capability, learning foundation, and confidence; one example is, “Learning AI-related skills is easy for me.”

#### 3.2.3 AI perceived usefulness

We adapted the perceived-usefulness scale of Achmadi et al. (2025), originally used to study AI adoption behavior. The wording was situated in students' learning and academic work because usefulness in this context concerns AI's contribution to learning tasks, productivity, and academic outcomes. The adapted four-item scale includes statements such as, “AI technology helps me achieve better academic performance.”

#### 3.2.4 AI learning anxiety

AI learning anxiety was measured with the learning-anxiety subscale of Wang and Wang (2022)'s artificial intelligence anxiety scale. Because the items already refer directly to learning AI, they were used without alteration. The eight items cover concerns about understanding functions, using and interacting with AI, taking AI-related courses, reading manuals, and keeping pace with technological updates. An example item is, “Learning to use AI technology makes me anxious.”

### 3.3 Analytical strategy

The data analysis followed a two-stage procedure. First, descriptive statistics and Harman's single-factor test were conducted using IBM SPSS Statistics (SPSS) (IBM Corp., Armonk, NY, USA). Reliability and validity were then assessed using Cronbach's alpha, composite reliability (CR), average variance extracted (AVE), standardized factor loadings, the Fornell-Larcker criterion, and the heterotrait-monotrait ratio (HTMT). CR, AVE, and discriminant-validity statistics were calculated from the standardized loadings and inter-construct correlations. Confirmatory factor analysis (CFA), SEM, mediation analysis, and the robustness model with control variables were estimated in Analysis of Moment Structures (AMOS). Bootstrap tests used 5,000 resamples and 95% bias-corrected confidence intervals (CIs). Because this observational study cannot establish causality, the SEM and bootstrap results should be interpreted as associations rather than causal effects. Second, a supplementary ANN predictive analysis was implemented in Python using scikit-learn. The ANN was estimated with the MLPRegressor class, one hidden layer with three neurons, hyperbolic tangent activation, the limited-memory Broyden-Fletcher-Goldfarb-Shanno (L-BFGS) optimizer, L2 regularization parameter α = 0.0001, max_iter = 5,000, and random_state = 20260716. Because L-BFGS is a full-batch quasi-Newton optimizer, learning-rate scheduling and mini-batch size were not applicable. A preliminary sensitivity check compared several small hidden-layer structures and optimizer-activation combinations; the final three-neuron tanh L-BFGS model was retained because it provided the best out-of-sample performance while remaining parsimonious. The ANN results are therefore reported as supplementary predictive evidence rather than as confirmatory causal evidence.

## 4 Results

### 4.1 Construct descriptive statistics and normality

**Figure 3:**
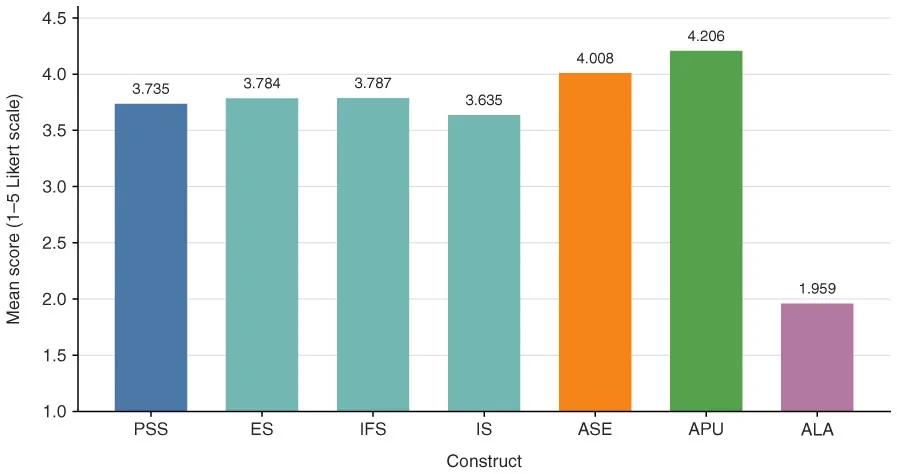
Mean scores of the main constructs.

Table 2 summarizes the main constructs. First-order construct scores were item means; thus, ES was obtained by averaging ES1–ES5 and ALA by averaging ALA1–ALA8. For the second-order PSS construct, scores for ES, IFS, and IS were first calculated and then averaged. Skewness ranged from −0.459 to 0.719 and kurtosis from −0.428 to 0.468, both within the conventional ±1 range, so no substantial departure from normality was evident. Mean (*M*) and standard deviation (SD) values indicated comparatively high levels of AI perceived usefulness (*M* = 4.206, SD = 0.603) and AI learning self-efficacy (*M* = 4.008, SD = 0.633); perceived school support was moderate to high (*M* = 3.735, SD = 0.738). AI learning anxiety was lower (*M* = 1.959, SD = 0.693), indicating that most respondents viewed AI learning as manageable and useful. Figure 3 visualizes these mean differences.

**Table 2:** Descriptive statistics of the main constructs.

Construct	Mean	SD	Min	Max	Skewness	Kurtosis
PSS	3.735	0.738	1.600	5.000	−0.193	−0.428
ES	3.784	0.777	1.600	5.000	−0.334	−0.285
IFS	3.787	0.807	1.250	5.000	−0.445	−0.103
IS	3.635	0.898	1.000	5.000	−0.323	−0.331
ASE	4.008	0.633	1.200	5.000	−0.409	0.224
APU	4.206	0.603	1.750	5.000	−0.459	−0.136
ALA	1.959	0.693	1.000	4.375	0.719	0.468

### 4.2 Assessment of common method bias

Because the study relied on self-report questionnaires, statistical checks were conducted to assess potential common method bias. Harman's single-factor test in SPSS showed that the first unrotated factor accounted for 37.078% of the total variance (Table 3), indicating that no single factor accounted for the majority of the variance among the measures. As a supplementary sensitivity analysis, a constrained equal-loading common latent factor (CLF) was specified in AMOS, with a unit method-factor variance and identical loadings across all 30 measurement items. Relative to the baseline model (χ^2^ = 566.894, df = 396, CFI = 0.980, TLI = 0.978, RMSEA = 0.028), the CLF model showed very similar fit (χ^2^ = 565.029, df = 395, CFI = 0.980, TLI = 0.978, RMSEA = 0.028; Δχ^2^ = 1.865, Δ df = 1, *p* = 0.172). The directions and statistical significance of the six substantive path estimates remained unchanged. However, these checks cannot fully rule out the possibility that common method bias affected the estimates.

**Table 3:** Total variance explained in Harman's single-factor test.

Component	Initial eigenvalues			Extraction sums of squared loadings		
	Total	Variance (%)	Cumulative (%)	Total	Variance (%)	Cumulative (%)
1	11.124	37.078	37.078	11.124	37.078	37.078
2	3.683	12.278	49.356	3.683	12.278	49.356
3	1.420	4.732	54.089	1.420	4.732	54.089
4	1.246	4.155	58.243	1.246	4.155	58.243
5	1.068	3.559	61.803	1.068	3.559	61.803
6	0.830	2.768	64.571			
7	0.669	2.231	66.802			
8	0.651	2.169	68.971			
9	0.630	2.099	71.070			
10	0.574	1.913	72.983			
11	0.548	1.827	74.810			
12	0.529	1.764	76.575			
13	0.512	1.708	78.283			
14	0.490	1.635	79.918			
15	0.477	1.589	81.506			
16	0.465	1.549	83.055			
17	0.462	1.540	84.595			
18	0.424	1.413	86.007			
19	0.414	1.381	87.388			
20	0.404	1.348	88.736			
21	0.395	1.317	90.054			
22	0.388	1.294	91.348			
23	0.376	1.255	92.603			
24	0.372	1.242	93.844			
25	0.352	1.173	95.018			
26	0.323	1.078	96.096			
27	0.321	1.071	97.167			
28	0.298	0.993	98.159			
29	0.281	0.937	99.097			
30	0.271	0.903	100.000			

### 4.3 Reliability and convergent validity assessment

Internal consistency and convergent validity were examined with alpha, CR, AVE, and standardized loadings. Table 4 reports CFA standardized regression weights; AVE was derived from their squared values. Alpha ranged from 0.823 to 0.909 and CR from 0.824 to 0.909, exceeding 0.70 in every case. AVE values ranged between 0.495 and 0.596. All constructs exceeded 0.50 except AI learning self-efficacy, whose AVE was only marginally lower. Item loadings were 0.668–0.791; although several did not reach 0.70, each exceeded 0.60. Taken together, these statistics support acceptable convergence.

**Table 4:** Reliability and convergent validity.

Construct	Item	Loading	AVE	CR	Cronbach's alpha
ALA	ALA1	0.720	0.556	0.909	0.909
	ALA2	0.768			
	ALA3	0.754			
	ALA4	0.753			
	ALA5	0.754			
	ALA6	0.769			
	ALA7	0.724			
	ALA8	0.719			
APU	APU1	0.729	0.539	0.824	0.823
	APU2	0.748			
	APU3	0.693			
	APU4	0.765			
ASE	ASE1	0.748	0.495	0.830	0.831
	ASE2	0.701			
	ASE3	0.705			
	ASE4	0.693			
	ASE5	0.668			
ES	ES1	0.727	0.549	0.859	0.861
	ES2	0.745			
	ES3	0.761			
	ES4	0.742			
	ES5	0.730			
IFS	IFS1	0.716	0.553	0.832	0.833
	IFS2	0.737			
	IFS3	0.781			
	IFS4	0.740			
IS	IS1	0.766	0.596	0.855	0.856
	IS2	0.791			
	IS3	0.775			
	IS4	0.755			

### 4.4 Discriminant validity assessment

Discriminant validity was evaluated with the Fornell–Larcker criterion and HTMT. Because PSS is a second-order construct formed by ES, IFS, and IS, the analysis used its first-order dimensions alongside the other focal latent variables. As reported in Table 5, the square root of AVE for every construct was larger than the corresponding absolute correlations. All HTMT values were below 0.90 (Table 6), indicating adequate construct distinctiveness.

**Table 5:** Fornell-Larcker discriminant validity assessment.

Construct	ALA	APU	ASE	ES	IFS	IS
ALA	**0.745**					
APU	−0.614	**0.734**				
ASE	−0.632	0.585	**0.703**			
ES	−0.414	0.407	0.362	**0.741**		
IFS	−0.444	0.387	0.372	0.696	**0.744**	
IS	−0.384	0.336	0.327	0.657	0.716	**0.772**

Bold diagonal values are the square roots of the average variance extracted (AVE) for their respective constructs; off-diagonal values are correlations between constructs.

**Table 6:** HTMT assessment of discriminant validity.

Construct	ALA	APU	ASE	ES	IFS	IS
ALA						
APU	0.710					
ASE	0.727	0.705				
ES	0.469	0.482	0.430			
IFS	0.510	0.467	0.447	0.823		
IS	0.436	0.401	0.389	0.766	0.850	

### 4.5 CFA measurement model fit

The measurement model was evaluated by CFA in AMOS. The indices in Table 7 supported an adequate fit: chi-square divided by degrees of freedom (χ^2^/df = 1.432), root mean square residual (RMR = 0.025), goodness-of-fit index (GFI = 0.936), adjusted goodness-of-fit index (AGFI = 0.925), comparative fit index (CFI = 0.980), Tucker–Lewis index (TLI = 0.978), and root mean square error of approximation (RMSEA = 0.028). All indices satisfied commonly used benchmarks.

**Table 7:** Fit indices for the measurement model.

Fit index	Estimate	Recommended threshold
χ^2^/df	1.432	<3.00
RMR	0.025	<0.05
GFI	0.936	>0.90
AGFI	0.925	>0.90
CFI	0.980	>0.90
TLI	0.978	>0.90
RMSEA	0.028	<0.08

### 4.6 Structural model and path coefficients

The structural model tested the proposed relations among PSS, ASE, APU, and ALA. Its fit indices matched those of the measurement model in Table 7. This equivalence follows from the fact that the four focal constructs allow six recursive paths, all of which were specified here: PSS → ASE, PSS → APU, PSS → ALA, ASE → APU, ASE → ALA, and APU → ALA. Consequently, the structural component has the same covariance structure as a freely correlated measurement model. The χ^2^, degrees of freedom, and fit indices are therefore unchanged and are not repeated.

**Figure 4:**
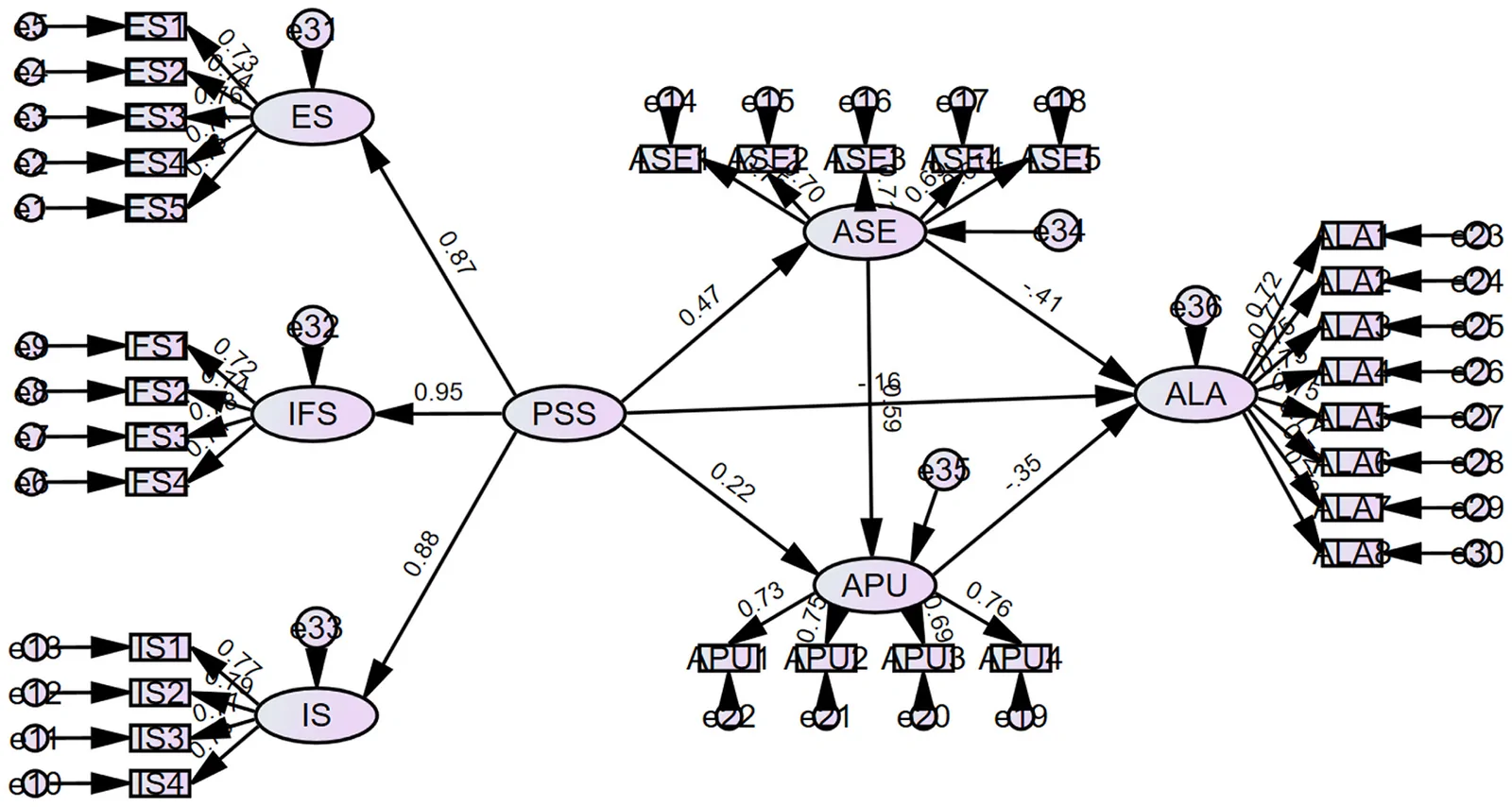
Standardized SEM results.

AMOS was used to evaluate the path estimates. Figure 4 and Table 8 present the standardized results. PSS positively predicted ASE (β = 0.469, *p* < 0.01) and APU (β = 0.223, *p* < 0.01); ASE also positively predicted APU (β = 0.587, *p* < 0.01). These findings support H2, H5, and H8, respectively. In contrast, ASE (β = −0.408, *p* < 0.01), APU (β = −0.345, *p* < 0.01), and PSS (β = −0.160, *p* < 0.01) each showed negative paths to ALA, supporting H3, H6, and H1, respectively. The squared multiple correlations were 0.220 for ASE, 0.517 for APU, and 0.623 for ALA, indicating that the structural model accounted for 22.0%, 51.7%, and 62.3% of the variance in these endogenous constructs, respectively. The relatively large explained variance in ALA indicates that, although the direct PSS–ALA association was modest, PSS, ASE, and APU jointly accounted for a substantial share of variation in AI learning anxiety.

**Table 8:** Standardized path coefficients of the structural model.

Path	Hyp.	β	95% CI lower	95% CI upper	*p*-Value
PSS → ALA	H1 (−)	−0.160	−0.248	−0.075	<0.01
PSS → ASE	H2 (+)	0.469	0.369	0.567	<0.01
ASE → ALA	H3 (−)	−0.408	−0.532	−0.277	<0.01
PSS → APU	H5 (+)	0.223	0.102	0.348	<0.01
APU → ALA	H6 (−)	−0.345	−0.483	−0.211	<0.01
ASE → APU	H8 (+)	0.587	0.452	0.707	<0.01

### 4.7 Bootstrap mediation and effect decomposition

AMOS mediation tests were conducted with user-defined estimands for the specific indirect paths. Table 9 shows a significant path from perceived school support to lower AI learning anxiety through AI learning self-efficacy [estimate = −0.207, 95% CI (−0.308, −0.136), *p* < 0.01], which supports H4. The indirect path through perceived usefulness was also significant [estimate = −0.083, 95% CI (−0.158, −0.034), *p* < 0.01], supporting H7. The ordered path via self-efficacy and then perceived usefulness was likewise significant [estimate = −0.103, 95% CI (−0.174, −0.060), *p* < 0.01], supporting H9. As detailed in Table 10, the unstandardized total effect was −0.566, comprising a direct effect of −0.173 and total indirect effect of −0.393; the latter represented 69.4% of the total. On the standardized scale, the total effect was −0.524, including −0.160 directly and −0.364 indirectly, with indirect effects accounting for 69.5%. Here, “direct effect” and “indirect effect” follow the standard terminology used in AMOS output. They are statistical labels and do not by themselves establish causality.

**Table 9:** Bootstrap test of specific indirect effects.

Specific indirect path	Hyp.	β	95% CI lower	95% CI upper	*p*-Value
PSS → ASE → ALA	H4 (−)	−0.207	−0.308	−0.136	<0.01
PSS → APU → ALA	H7 (−)	−0.083	−0.158	−0.034	<0.01
PSS → ASE → APU → ALA	H9 (−)	−0.103	−0.174	−0.060	<0.01
Total indirect effect	–	−0.393	–	–	–

**Table 10:** Decomposition of the effect of perceived school support on AI learning anxiety.

Effect component	Unstandardized effect		Standardized effect	
	Estimate	Share of total	Estimate	Share of total
Direct effect	−0.173	30.6%	−0.160	30.5%
Indirect effect	−0.393	69.4%	−0.364	69.5%
Total effect	−0.566	100.0%	−0.524	100.0%

### 4.8 Supplementary first-order support-dimension model

To assess whether the conclusions depended on representing perceived school support as a second-order construct, a supplementary first-order model was estimated. ES, IFS, and IS were allowed to covary and were separately linked to ASE, APU, and ALA, while the paths among ASE, APU, and ALA were retained. The model fit the data well (χ^2^ = 559.000, *df* = 390, χ^2^/*df* = 1.433, CFI = 0.980, TLI = 0.978, RMSEA = 0.028). As shown in Table 11, after accounting for the shared variance among the three support dimensions, only the IFS–ASE path retained a statistically significant BC bootstrap interval [β = 0.288, 95% CI (0.052, 0.558), *p* = 0.020]. The remaining dimension-specific paths had confidence intervals that included zero. Thus, the first-order analysis provided limited evidence of distinct dimension-specific linear associations with the focal constructs and showed no material improvement in the reported fit indices relative to the second-order model despite estimating additional paths. The second-order PSS representation was therefore retained in the main SEM as a parsimonious representation of overall perceived school support and the variance shared across its three dimensions.

**Table 11:** Standardized paths in the first-order support-dimension model.

Path	β	95% CI lower	95% CI upper	*p*-Value
ES → ASE	0.207	−0.010	0.412	0.059
IFS → ASE	0.288	0.052	0.558	0.020
IS → ASE	−0.013	−0.266	0.205	0.906
ES → APU	0.203	−0.010	0.428	0.062
IFS → APU	0.088	−0.184	0.359	0.512
IS → APU	−0.057	−0.287	0.169	0.622
ES → ALA	0.051	−0.157	0.236	0.619
IFS → ALA	−0.213	−0.476	0.047	0.104
IS → ALA	0.008	−0.193	0.212	0.994

### 4.9 Robustness analysis with control variables

To examine whether the main findings were sensitive to demographic and usage-related differences, gender, major, and AI tool use frequency were added as control variables in the AMOS structural model. The control variables were specified as exogenous observed variables predicting AI learning self-efficacy, AI perceived usefulness, and AI learning anxiety. The model with controls continued to show good fit: χ^2^/df = 1.414, *RMR* = 0.024, *GFI* = 0.932, *AGFI* = 0.919, *CFI* = 0.977, *TLI* = 0.974, and *RMSEA* = 0.027. As shown in Table 12, the signs and significance levels of the main structural paths remained substantively unchanged after controlling for gender, major, and AI tool use frequency. In addition, no significant direct effects were found from the control variables to the three endogenous constructs.

**Table 12:** Robustness check with control variables.

Path	Main model β	Model with controls β (95% CI)	*p*-Value
Main structural paths			
PSS → ASE	0.469	0.465 (0.365, 0.565)	<0.01
PSS → APU	0.223	0.217 (0.096, 0.340)	<0.01
ASE → APU	0.587	0.586 (0.451, 0.704)	<0.01
ASE → ALA	−0.408	−0.411 (−0.537, −0.279)	<0.01
APU → ALA	−0.345	−0.339 (−0.478, −0.207)	<0.01
PSS → ALA	−0.160	−0.158 (−0.247, −0.074)	<0.01
Control paths			
Frequency → ASE	–	0.005 (−0.072, 0.092)	0.882
Major → ASE	–	0.053 (−0.033, 0.134)	0.236
Gender → ASE	–	−0.072 (−0.157, 0.017)	0.105
Frequency → APU	–	0.009 (−0.060, 0.083)	0.756
Major → APU	–	−0.044 (−0.116, 0.035)	0.259
Gender → APU	–	−0.063 (−0.147, 0.013)	0.104
Frequency → ALA	–	0.021 (−0.040, 0.078)	0.496
Major → ALA	–	0.018 (−0.049, 0.086)	0.583
Gender → ALA	–	0.038 (−0.024, 0.105)	0.247

### 4.10 Supplementary ANN prediction and predictor importance

Following the SEM hypothesis tests, we fitted an ANN as a supplementary predictive model with AI learning anxiety as the outcome. SEM addressed theory-based directional relations among latent constructs, whereas the ANN assessed out-of-sample prediction and the relative importance of predictors. The ANN findings should therefore be viewed as predictive evidence that complements the theoretically specified directional associations examined in SEM; neither analysis establishes causal direction.

PSS was represented as a higher-order construct in SEM, whereas the ANN separated it into its three first-order dimensions to assess their individual predictive contributions. The inputs were ES, IFS, IS, ASE, and APU. A 5–3–1 network was specified: five input neurons, three hidden neurons that learned non-linear combinations, and one output neuron for ALA. The hidden layer used a hyperbolic tangent activation function, and training minimized mean squared prediction error. The model is written as follows:


$$\begin{array}{l} \hat{ALA}=f\left(ES,IFS,IS,ASE,APU\right), \end{array}$$

where *f*(·) is the non-linear function learned from the five inputs to predict AI learning anxiety. To avoid relying on a single train–test partition, we used repeated 10-fold cross-validation with 10 repetitions, yielding 100 held-out evaluations. Each fold trained the model on 90% of cases and tested it on the remaining 10%. Inputs were standardized within the training data, and the same transformation was applied to its corresponding test data. These 100 evaluations represent resampling-based internal cross-validation evidence rather than 100 independent external validation samples. Held-out coefficient of determination (*R*^2^) was computed as one minus the residual-to-total sum-of-squares ratio. We estimated a mean-only model, ordinary linear regression, and Ridge regression (α = 1.0) on the same folds as benchmarks.

Predictor importance was evaluated on the held-out test folds with a permutation procedure. After the trained ANN produced its baseline error, one input was shuffled while the remaining inputs were unchanged. A larger rise in prediction error indicated a greater contribution to prediction. The raw importance score was the increase in mean squared error (MSE):


$$\begin{array}{l} I_{j}=MSE\left({\tilde{X}}_{j}\right)-MSE\left(X\right), \end{array}$$

where *MSE*(*X*) is the baseline test error and $MSE\left({\tilde{X}}_{j}\right)$ is the test error after randomly permuting predictor *j*. To make the scores easier to compare, the raw importance values were normalized as a relative importance index (RI):


$$\begin{array}{l} RI_{j}=\frac{I_{j}}{\max\left(I_{1},I_{2},\ldots ,I_{p}\right)}\times 100. \end{array}$$

An importance value of 100 identifies the input whose permutation produced the greatest error increase. It is not a percentage of explained AI learning anxiety, and normalized scores need not total 100. Predictive performance was reported using root mean squared error (RMSE), mean absolute error (MAE), and held-out *R*^2^.

As shown in Table 13, the ANN achieved a mean testing RMSE of 0.4742, a mean testing MAE of 0.3555, and a mean testing *R*^2^ of 0.5126 across the 100 held-out evaluations, indicating a moderate level of out-of-sample predictive performance for AI learning anxiety. It substantially outperformed the mean-only baseline and showed modestly better average predictive accuracy than both the ordinary linear regression and Ridge regression benchmarks. Compared with linear regression, the ANN reduced mean testing RMSE by 0.0096 and MAE by 0.0073, while increasing mean testing *R*^2^ by 0.0183. Thus, the improvement over the linear benchmarks was modest.

**Table 13:** Predictive performance of the ANN model and benchmark models.

Model	Testing RMSE (*M*, SD)	Testing MAE (*M*, SD)	Testing *R*^2^ (*M*, SD)
ANN	0.4742 (0.0543)	0.3555 (0.0427)	0.5126 (0.1045)
Linear regression	0.4838 (0.0546)	0.3628 (0.0406)	0.4942 (0.1019)
Ridge regression	0.4837 (0.0546)	0.3628 (0.0406)	0.4943 (0.1018)
Mean baseline	0.6912 (0.0584)	0.5372 (0.0490)	−0.0177 (0.0215)

**Figure 5:**
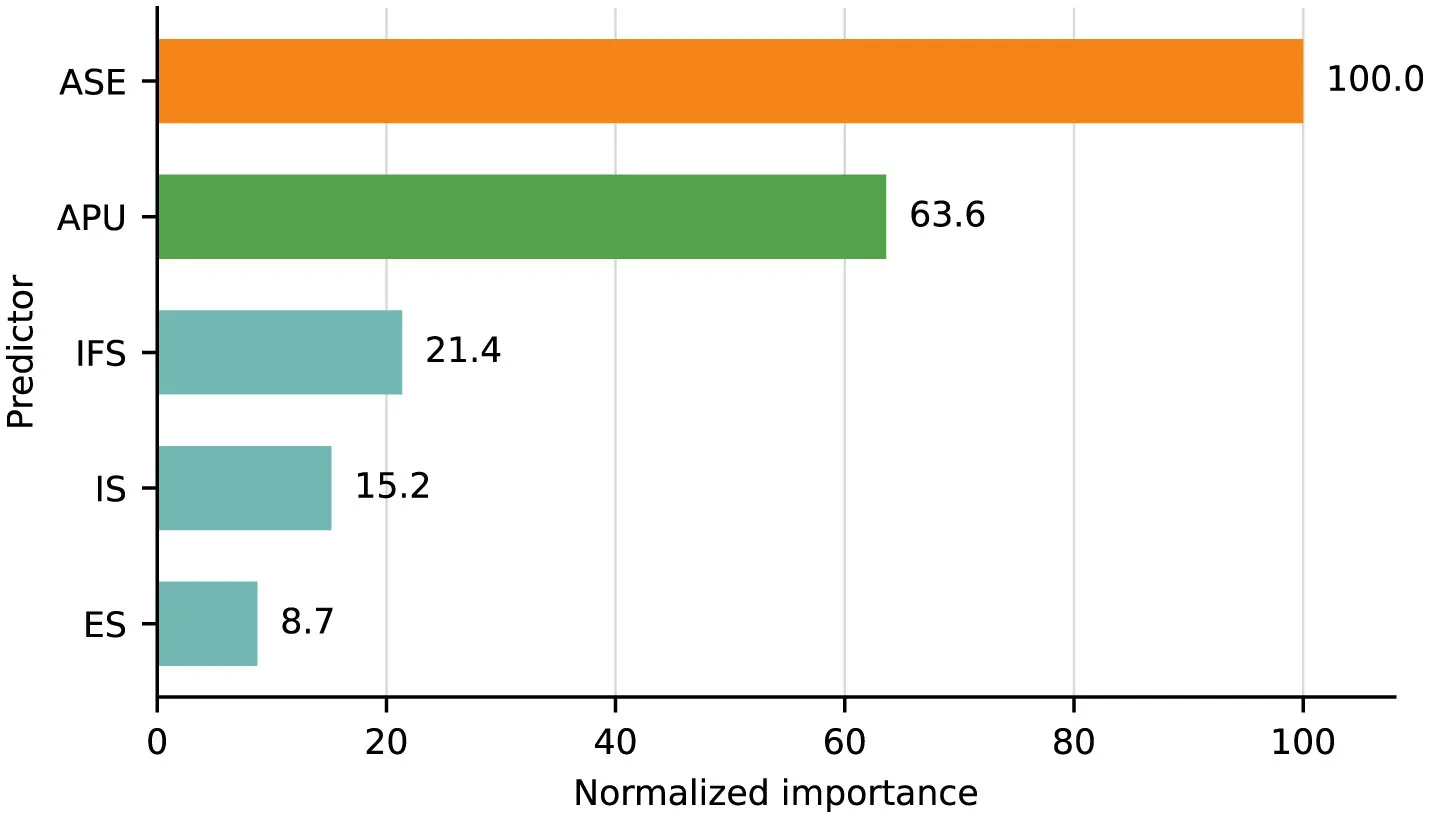
Normalized predictor importance from the ANN model for AI learning anxiety.

As shown in Table 14 and Figure 5, AI learning self-efficacy was the most important predictor of AI learning anxiety in the ANN model, followed by AI perceived usefulness. Among the three perceived-school-support dimensions, informational support had the largest mean normalized importance, followed by instrumental support and emotional support. However, the standard deviations of the three support-related importance scores were similar to or larger than their mean values. These findings should therefore be interpreted cautiously. They indicate that the support dimensions contained some predictive information when examined separately, but their ANN importance scores were less stable than those of AI learning self-efficacy and AI perceived usefulness. These importance values are relative predictive importance scores, not causal effects or proportions of explained variance.

**Table 14:** Predictor importance in the ANN model.

Predictor	Permutation importance	SD	Normalized importance
ASE	0.1660	0.0682	100.0
APU	0.1056	0.0537	63.6
IFS	0.0355	0.0410	21.4
IS	0.0252	0.0342	15.2
ES	0.0145	0.0271	8.7

Taken together, the cross-validated ANN results showed only a small predictive advantage over the linear and Ridge-regression benchmarks. This pattern suggests that the linear relations represented in SEM captured most of the predictive information in the data, while ANN provides supplementary predictive evidence consistent with the SEM pattern. SEM represented PSS as a higher-order construct and examined the indirect associations of PSS with ALA through ASE and APU. In contrast, ANN did not impose this structural ordering: it entered ES, IFS, IS, ASE, and APU simultaneously as predictors of ALA. This enabled a dimension-level examination of which forms of school support contributed predictive information beyond the two appraisal variables. The analysis identified ASE and APU as the strongest and most stable predictors of ALA. Among the three school-support dimensions, informational support showed the largest average predictive contribution, whereas the support-dimension importance estimates were less stable than those for ASE and APU. Thus, ANN complements SEM by identifying the relative predictive relevance of individual support dimensions, but it does not test mediation or establish causal directions.

## 5 Discussion

### 5.1 Summary of main findings

The SEM findings showed that Chinese university students who perceived more school support reported less AI learning anxiety. Perceived school support was positively related to AI learning self-efficacy and perceived usefulness, while both appraisals were negatively related to anxiety. Every hypothesized direct association was supported in its predicted direction.

Two significant specific indirect associations were observed, one involving AI learning self-efficacy and the other involving perceived usefulness. A significant sequential indirect association involving both appraisals in the theoretically specified order was also observed. These indirect associations represented a larger proportion of the modeled total association than the direct association. The findings were consistent with the proposed mediation model but do not establish temporal ordering or causal mediation.

The robustness model produced substantively similar structural estimates after gender, academic major, and AI tool use frequency were controlled. The supplementary ANN analysis also achieved moderate out-of-sample prediction and ranked AI learning self-efficacy and AI perceived usefulness as the strongest predictors of AI learning anxiety. By contrast, the separate support dimensions had weaker and less stable predictive importance. Together, these checks indicate that the main SEM pattern was stable within the present sample and that the two appraisals were especially relevant to prediction.

Taken together, the SEM and ANN findings identify AI learning self-efficacy and perceived usefulness as central correlates of AI learning anxiety within this sample. This pattern can be considered alongside studies from Turkey, South Korea, and Saudi Arabia that relate AI anxiety to individual resources, learning practices, and motivation (Cengiz and Peker, 2025; Kim, 2026; Mohamed et al., 2026). Whereas those studies primarily focus on individual-level correlates, the present study additionally situates these appraisals in an educational context by examining perceived school support and its associations with self-efficacy, perceived usefulness, and AI learning anxiety.

### 5.2 Theoretical implications

This study broadens work on AI learning anxiety by examining perceived school support as a contextual factor associated with AI learning anxiety. Previous research has often approached anxiety as a correlate or predictor of learning behavior, intention, and motivation, or emphasized students' AI literacy and self-efficacy (Wang Y.-M. et al., 2024; Chen et al., 2025; Karaoglan Yilmaz et al., 2025). Our results extend this individual-level focus by treating perceived school support as a contextual condition associated with emotional responses to AI-supported learning. This perspective highlights the relevance of university support not only to students' access to AI but also to the psychological conditions associated with learning to use it.

This pattern is consistent with an appraisal-based account in which the association between school support and anxiety may reflect students' perceptions of the controllability and value of AI-related tasks. Clear institutional expectations, examples of appropriate use, and timely feedback can reduce uncertainty about competent performance. Students may therefore be less likely to attribute an initially poor prompt, an inaccurate output, or difficulty operating a tool to a stable lack of ability. Such a shift is important because perceived control is a central antecedent of achievement emotions in CVT (Pekrun, 2006, 2024). Low-stakes practice, guided comparison of AI outputs, and opportunities to revise unsuccessful attempts can also provide mastery experiences that strengthen AI learning self-efficacy (Bandura, 1997). The observed pattern is consistent with anxiety being related to perceived control and task value, with stronger indirect associations through self-efficacy and perceived usefulness than through the direct path.

The results also offer an integrated account of the role of control and value. In CVT terms, AI learning self-efficacy represents perceived control over AI-related tasks (Pekrun, 2006); in TAM, perceived usefulness captures the instrumental value assigned to AI for academic work (Davis, 1989). The observed indirect associations are consistent with AI learning anxiety being related not only to unfamiliar technology but also to whether students believe they can manage AI learning and whether they consider AI worthwhile for their academic work. The significant serial indirect association was also consistent with a connection between control and value: greater perceived school support was associated with higher AI learning self-efficacy, higher self-efficacy with greater perceived usefulness, and greater perceived usefulness with lower AI learning anxiety. This account is compatible with CVT and with AI-adoption research reporting a positive association between self-efficacy and perceived usefulness (Pekrun, 2006; Shao et al., 2025).

The findings can also be placed within the broader educational aim of psychological literacy in AI-supported learning. Psychological literacy emphasizes the application of psychological knowledge and reflective judgment when engaging with AI, rather than reducing effective AI use to technical competence alone (Roberts et al., 2015; Cranney et al., 2022). In the present sample, perceived school support was associated with more favorable control and value appraisals and lower AI learning anxiety. This pattern is consistent with the view that universities can support psychologically literate engagement with AI not only by providing access to tools, but also by helping students understand their perceived control over AI-related learning tasks, make informed judgments about AI's educational value, and manage the anxiety that may arise in AI-supported learning. Emotional encouragement, clear guidance, and practical resources may provide the educational conditions for this process.

### 5.3 Practical implications

The observed associations highlight the potential relevance of emotional, informational, and instrumental school support for addressing AI learning anxiety. Based on these associations, universities could operationalize these forms of support through concrete institutional strategies, particularly by developing responsible AI use guidelines and establishing accessible technical-support mechanisms. The guidelines should specify appropriate uses, disclosure requirements, verification of AI-generated content, privacy protection, and academic-integrity expectations (Chan, 2023; Cotton et al., 2024). To support the implementation of these guidelines, universities should establish visible technical-support mechanisms that provide access to institutionally approved AI tools, necessary devices and digital resources, help desks, and troubleshooting services.

The observed indirect associations involving AI learning self-efficacy and perceived usefulness suggest that university initiatives could focus on undergraduates' confidence in using AI and their recognition of its value in education. Universities could offer AI literacy courses or training and draw on existing technological resources and platforms to guide undergraduates in the appropriate use of AI for learning (Chan, 2023). Corresponding technical and pedagogical training could also strengthen faculty members' capacity to guide undergraduates in AI-supported learning (Laru et al., 2025).

Universities can also embed school support in AI-supported learning activities through explicit assessment criteria, instructor guidance, and feedback. For example, in an undergraduate psychology course, students evaluated AI-generated content against assessment criteria, identified and corrected inaccuracies, and applied feedback. Such activities can integrate the development of AI, feedback, and psychological literacies (Richmond and Nicholls, 2025). They give students repeated practice in judging the quality and educational value of AI outputs, revising their evaluations in response to feedback, and reflecting on their cognitive and emotional experiences in AI-supported learning. In this way, they can help students develop a more informed sense of control over AI-related learning tasks, make reasoned judgments about AI's educational value, and recognize and manage anxiety arising during AI-supported learning. Adapted and evaluated across disciplinary contexts, similar activities could complement students' technical knowledge and practical AI skills with opportunities for critical and reflective engagement with AI. These initiatives may contribute to a more supportive AI-learning environment characterized by greater student confidence in using AI, stronger recognition of its educational value, and lower AI learning anxiety.

### 5.4 Limitations and future research

Several limitations should be noted. First, despite the temporal separation of some measures, the present design did not meet the requirements for a longitudinal study and should therefore be interpreted as a cross-sectional structural association analysis. It cannot establish temporal precedence, causal effects, or causal mediation, or rule out alternative or reciprocal relationships and unmeasured confounding. Future research should use properly designed multi-wave longitudinal studies to clarify temporal ordering and reciprocal relationships, as well as experimental or quasi-experimental designs to test causal effects.

Second, the sample consisted exclusively of students from Chinese universities. This convenience sample included only students with prior AI-tool experience, as the questionnaire required experience-based evaluations of AI-supported learning. The findings are therefore most directly applicable to students with comparable experience. AI learning anxiety was also generally low and showed limited variability, which may have affected the magnitude of the estimated associations. Although controlling for gender, major, and AI-use frequency did not materially alter the main paths, the uneven distribution of these characteristics in this convenience sample warrants caution when generalizing the findings. The findings may therefore reflect features of the Chinese higher education context and should not be generalized directly to universities in other countries or cultural and educational settings. Generalization to students with limited or no AI experience also requires caution. Future studies should examine whether the observed associations can be replicated across countries, institution types, and more diverse student populations.

Third, the study relied entirely on self-report questionnaires. The responses may therefore have been affected by response styles, recall limitations, social-desirability concerns, and common method bias. Moreover, the study did not include objective indicators of AI competence or academic performance, making it impossible to determine whether students' reported beliefs and anxiety corresponded to their actual AI skills or learning outcomes. Future studies should combine self-reports with performance-based AI assessments, behavioral measures, or academic records.

## 6 Conclusion

This study examined the association between perceived school support and AI learning anxiety among undergraduates enrolled at Chinese universities. Greater perceived school support was associated with higher AI learning self-efficacy and perceived usefulness, both of which were associated with lower AI learning anxiety. Two significant specific indirect associations, one through each appraisal, and a significant sequential indirect association involving both appraisals were observed between perceived school support and AI learning anxiety. These findings were consistent with the proposed mediation model but do not establish causal mediation.

The principal theoretical contribution of this study is to integrate Control-Value Theory and the Technology Acceptance Model to interpret AI learning anxiety. Control-Value Theory conceptualizes this anxiety as an achievement emotion associated with control and value appraisals, whereas the Technology Acceptance Model specifies perceived usefulness as a technology-specific value appraisal centered on AI's utility for learning. Integrating the two frameworks extends the role of perceived usefulness beyond technology acceptance and places it alongside AI learning self-efficacy, conceived as a control appraisal, in a common framework for interpreting their associations with AI learning anxiety. Social Support Theory provides the educational context by framing perceived school support as a contextual correlate of these appraisals. Together, these perspectives provide a theory-guided framework for interpreting the observed associations among perceived school support, students' control and value appraisals, and AI learning anxiety in AI-supported learning.

Building on this integrated framework, universities should foster supportive AI-learning environments by embedding emotional encouragement, learning guidance, and technological resources into sustained opportunities for practice and feedback. Universities should also establish clear guidelines for AI use and provide appropriate instructional support to help university students recognize the educational value of AI and use it responsibly. These efforts may strengthen university students' confidence in AI-supported learning, help them adapt to the demands of learning in the AI era, and reduce anxiety associated with technological uncertainty and perceived lack of competence. In the longer term, continued improvement in university-level AI support may contribute to more inclusive learning environments and support university students' academic development and broader skill development.

## Data Availability

The original contributions presented in the study are included in the article/supplementary material, further inquiries can be directed to the corresponding authors.
